# Prognostic importance of numbers of retrieved lymph nodes and positive lymph nodes for ampulla of vater cancer (AVC) in 2347 patients from the Surveillance, Epidemiology, and End Results (SEER) database

**DOI:** 10.1371/journal.pone.0244987

**Published:** 2021-01-15

**Authors:** Jiao Feng, RuiYang Wu, Gang Zhang, ZhiMing Yang, Liang Zhang

**Affiliations:** 1 Department of Gastrointestinal Surgery, The Affiliated Chengdu 363 Hospital of Southwest Medical University, Chengdu, Sichuan, China; 2 Department of General Surgery, Sichuan Provincial Hospital for Women and Children (Affiliated Women and Children’s Hospital of Chengdu Medical College), Chengdu, Sichuan, China; 3 Department of Vascular Surgery, The First Affiliated Hospital of Chengdu Medical College, Chengdu, Sichuan, China; University of Maryland, UNITED STATES

## Abstract

**Introduction:**

The numbers of retrieved lymph nodes (RLNs) and positive lymph nodes (PLNs) had a significant impact on the survival of patients with ampulla of vater cancer (AVC), but the optimal numbers of the both were controversial.

**Objective:**

The cohort study aimed to explore the prognostic value and the optimal point of RLNs and PLNs for AVC.

**Methods:**

A total of 2347 AVC patients with M0 disease who underwent surgical resection and lymph node dissection from January 2004 to December 2013 were acquired from a prospective database.

**Results:**

The study found that the optimal cut-off values of RLNs were 18 in the N0 cohort and 16 in N1 or entire cohort due to the highest 5-year overall survival (OS) rate and disease-specific survival (DSS) rate and the separation of survival curves (all P < 0.05). In patients with RLNs ≥ 16, patients with PLN = 0 demonstrated significantly better 5-year OS and DSS rates (70.9% and 77.1%) compared to those with PLNs = 1–2 (41.6% and 44.7%; all P < 0.001), and patients with PLNs = 1–2 demonstrated significantly better 5-year OS and DSS rates (41.6% and 44.7%) compared to those with PLNs ≥ 3 (24.3% and 28.0%; all P < 0.001).

**Conclusions:**

This article recommended that at least 16 lymph nodes will improve the prognosis of AVC patients undergoing surgery. The best cut-off values of PLNs recommended for this study were 0 and 2, which may accurately stratify patients.

## Introduction

Ampulla of vater cancer (AVC) was a rare malignancy arising from the papilla of vater [1], and the incidence of AVC in the United States has increased year by year since 1973 [2]. Among periampullary adenocarcinoma, the prognosis of AVC was better than pancreatic head cancer or bile duct cancer [3]. However, lymph node metastasis may exist even in early ampullary carcinoma [4], and the rate of lymph node metastasis has been reported as high as 31.3–58.8% [5–7]. Therefore, lymph node dissection was recommended during surgical resection [8]. Increasing evidence indicated that the numbers of retrieved lymph nodes (RLNs) and positive lymph nodes (PLNs) had a significant impact on the survival of patients with AVC [9–12]. Due to the relative lack of disease, current studies about the effects of RLNs and PLNs on the prognosis of patients with AVC were mostly small sample sizes [9, 12–14], and the optimal numbers of the both were controversial. Thus, the cohort study aimed to explore the prognostic value of RLNs and PLNs for AVC and to determine the optimal point of RLNs and PLNs at the population level through a national database.

## Materials and methods

### Database and samples

The data from the Surveillance, Epidemiology, and End Results (SEER) database was acquired from the SEER*Stat 8.3.6 Software (https://seer.cancer.gov/data/), and we accessed the SEER database on March 1, 2020. A total of 2875 AVC patients who underwent surgical resection and lymph node dissection (RLNs ≥ 1) from January 2004 to December 2013 were identified by that primary site of tumor was C24.1-Ampulla of Vater, year of diagnosis was from 2004 to 2013, surgery of primary tumor was encoded from 20 to 90 (such as simple/partial surgical removal of primary site, total surgical removal of primary site, radical surgery), number of lymph nodes examined ≥ 1, diagnostic confirmation was positive histology, and type of follow-up expected was active follow-up. A number of 510 patients who < 18 years old or > 80 years old, died within 1 month, and had M1, MX, or NX diseases were excluded. Finally, 2347 AVC patients with M0 disease were included in the study.

The data collected in this study included age at diagnosis, gender, race, marital status at diagnosis, tumor size, tumor grade, lymph node metastasis, number of RLNs and PLNs, adjuvant radiotherapy and chemotherapy.

### Statistical analysis

Continuous variables and categorical variables were presented as median (range) and frequency (percentage). Overall survival (OS) and disease-specific survival (DSS) rates were calculated from diagnosis to death due to any reason and from diagnosis to death due to VAC, respectively. The last follow-up time was November 2018. To get the optimal cut-off value of RLNs, the survival analysis performed by Kaplan-Meier method (log-rank test) was compared the 5-year OS and DSS rates of different cut-off values of RLNs in N0, N1 and entire cohorts, respectively, and it was used the 5-year survival rates and P values of different cut-off values of RLNs as line charts. The optimal number of PLNs was gained by X-tile software (Version 3.6.1, Yale University) and verified by the survival analysis. A two-tailed P < 0.05 was considered statistically significant. Statistical analysis was using STATA 16.0 software. Ethics statement was not required for this study, because SEER database was publicly available. In addition, Data-Use Agreements for the 1975–2017 SEER Research Data File and SEER Radiation Therapy and Chemotherapy Information were signed and the database can be accessed.

## Results

### Baseline characteristics

During the period from January 2004 to December 2013, 2347 AVC patients with M0 disease who underwent surgical resection and lymph node dissection from the SEER database were included in this study (**Table 1**). The median age at diagnosis was 65 (20–80) years, and male accounted for about 56.8% (1333/2347). The vast majority (78.7%) were white, 7.3% (172/2347) blcak, and 13.9% (327/2347) other. A total of 33255 lymph nodes were examined with a median number of 13 (1–67), and a total of 4105 positive lymph nodes were found with a median number of 1 (0–31). Tumor size for 145 (6.2%) patients was unknown, and the median tumor size for the remaining patients (93.8%) was 21 (1–87) mm. The median follow-up was 39 (2–155) months. As of November 2018, 981 (41.8%) patients were alive and 1366 (58.2%) patients had died. In the entire cohort (n = 2347), the 1-, 3-, and 5-year OS rates were 85.1%, 56.4%, and 45.5%, respectively, and the corresponding DSS rates were 86.8%, 60.0%, and 50.8%, respectively.

**Table 1:** Demographics and clinical characteristics for AVC patients with M0 disease (n = 2347).

Demographics	Ampulla of vater cancer (n = 2347)
Age at diagnosis (years)	65(20–80)
Gender, male/female (%)	1333(56.8)/1014(43.2)
Race, n (%)	
white	1848(78.7)
black	172(7.3)
other	327(13.9)
Marital status at diagnosis, n (%)	
married	1509(64.3)
single/widowed/divorced	751(32.0)
unkown	87(3.7)
Tumor size (mm)	21(1–120)
Tumor grade, n (%)	
well differentiated	272(11.6)
moderately differentiated	1206(51.4)
poorly differentiated	741(31.6)
undifferentiated	19(0.8)
unkown	109(4.6)
Lymph node metastasis, yes/no (%)	1211(51.6)/1136(48.4)
Number of retrieved lymph nodes	13(1–67)
Number of positive lymph nodes	1(0–31)
Adjuvant radiotherapy, yes/no or unkown (%)	609(25.9)/1738(74.1)
Adjuvant chemotherapy, yes/no or unkown (%)	1073(45.7)/1274(54.3)

The other comprises American Indian/Alaska Native, Asian/Pacific Islander.

### Exploration for the optimal cut-off value of retrieved lymph nodes

**Fig 1:**
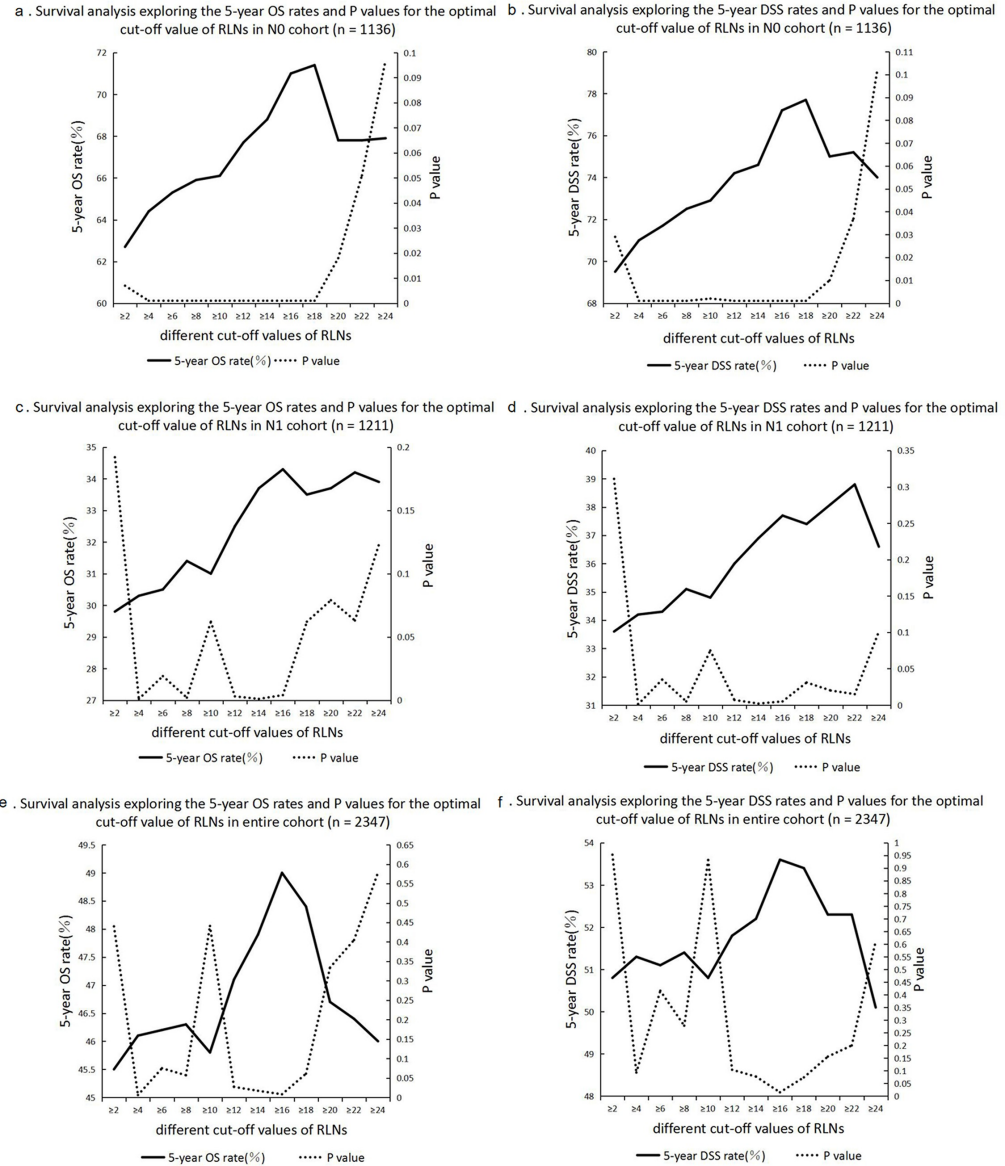
Survival analysis comparing the different numbers of PLNs. a: Survival analysis exploring the 5-year OS rates and P values for the optimal cut-off value of RLNs in N0 cohort (n = 1136). b: Survival analysis exploring the 5-year DSS rates and P values for the optimal cut-off value of RLNs in N0 cohort (n = 1136). c: Survival analysis exploring the 5-year OS rates and P values for the optimal cut-off value of RLNs in N1 cohort (n = 1211). d: Survival analysis exploring the 5-year DSS rates and P values for the optimal cut-off value of RLNs in N1 cohort (n = 1211). e: Survival analysis exploring the 5-year OS rates and P values for the optimal cut-off value of RLNs in entire cohort (n = 2347). f: Survival analysis exploring the 5-year DSS rates and P values for the optimal cut-off value of RLNs in entire cohort (n = 2347).

For exploring the optimal cut-off value of RLNs, survival analysis was conducted to compared the 5-year OS and DSS rates of different cut-off values of RLNs in N0, N1 and entire cohorts for AVC patients with M0 disease was compared by survival analysis (**Table 2**), and it was used the 5-year survival rates and P values of different cut-off values of RLNs as line charts (**Fig 1**). In N0 cohort (n = 1136), the optimal cut-off value of RLNs was 18 due to the highest 5-year OS rate (71.4%) and DSS rate (77.7%) and separation of survival curves between patients with RLNs ≥ 18 and RLNs < 18 (**Fig 1A** and **1B**; all P < 0.001). In N1 cohort (n = 1211), the optimal cut-off value of RLNs was 16 due to the highest 5-year OS rate (34.3%) and the third highest 5-year DSS rate (37.7%) and separation of survival curves between patients with RLNs ≥ 16 and RLNs < 16 (**Fig 1C**, P = 0.004; **Fig 1D**, P = 0.005). In the entire cohort (n = 2347), the optimal cut-off value of RLNs was 16 due to the highest 5-year OS rate (49.0%) and DSS rate (53.6%) and separation of survival curves between patients with RLNs ≥ 16 and RLNs < 16 (**Fig 1E**, P = 0.008; **Fig 1F**, P = 0.015).

**Table 2:** Survival analysis comparing the 5-year OS and DSS rates of different cut-off values of RLNs in N0, N1 and entire cohorts for AVC patients with M0 disease (n = 2347).

Retrieved lymph nodes	N	Overall Survival (OS)		Disease-specific survival (DSS)	
		5-year OS rate (%)	P value	5-year DSS rate (%)	P value
**In N0 cohort**	1136				
< 2	44	49.8	0.007	57.8	0.029
≥ 2	1092	62.7		69.5	
< 4	136	46.9	<0.001	55.1	<0.001
≥ 4	1000	64.4		71.0	
< 6	241	51.1	<0.001	59.5	0.001
≥ 6	895	65.3		71.7	
< 8	361	54.4	<0.001	61.7	<0.001
≥ 8	775	65.9		72.5	
< 10	479	57.0	<0.001	63.9	0.002
≥ 10	657	66.1		72.9	
< 12	585	57.1	<0.001	64.3	<0.001
≥ 12	551	67.7		74.2	
< 14	692	58.2	<0.001	65.6	<0.001
≥ 14	444	68.8		74.6	
< 16	785	58.4	<0.001	65.5	<0.001
≥ 16	351	71.0		77.2	
< 18	862	59.4	<0.001	66.4	<0.001
≥ 18	274	71.4		77.7	
< 20	929	61.0	0.018	67.8	0.010
≥ 20	207	67.8		75.0	
< 22	987	61.4	0.051	68.2	0.037
≥ 22	149	67.8		75.2	
< 24	1030	61.6	0.097	68.6	0.102
≥ 24	106	67.9		74.0	
**In N1 cohort**	1211				
< 2	7	14.3	0.192	19.0	0.311
≥ 2	1204	29.8		33.6	
< 4	48	15.1	0.001	18.7	0.001
≥ 4	1163	30.3		34.2	
< 6	113	21.7	0.019	27.3	0.035
≥ 6	1098	30.5		34.3	
< 8	198	21.3	0.002	25.5	0.005
≥ 8	1013	31.4		35.1	
< 10	304	25.7	0.062	29.9	0.075
≥ 10	907	31.0		34.8	
< 12	434	24.9	0.003	29.3	0.007
≥ 12	777	32.5		36.0	
< 14	557	25.2	0.001	29.8	0.002
≥ 14	654	33.7		36.9	
< 16	684	26.4	0.004	30.5	0.005
≥ 16	527	34.3		37.7	
< 18	787	27.8	0.062	31.5	0.031
≥ 18	424	33.5		37.4	
< 20	877	28.2	0.079	31.9	0.020
≥ 20	334	33.7		38.1	
< 22	951	28.5	0.063	32.1	0.015
≥ 22	260	34.2		38.8	
< 24	1019	28.9	0.123	33.0	0.101
≥ 24	192	33.9		36.6	
**In entire cohort**	2347				
< 2	51	44.9	0.440	52.6	0.953
≥ 2	2296	45.5		50.8	
< 4	184	38.6	0.006	45.6	0.093
≥ 4	2163	46.1		51.3	
< 6	354	41.7	0.075	49.2	0.416
≥ 6	1993	46.2		51.1	
< 8	559	42.8	0.057	49.1	0.278
≥ 8	1788	46.3		51.4	
< 10	783	44.9	0.441	50.9	0.932
≥ 10	1564	45.8		50.8	
< 12	1019	43.4	0.027	49.6	0.104
≥ 12	1328	47.1		51.8	
< 14	1249	43.4	0.017	49.7	0.077
≥ 14	1098	47.9		52.2	
< 16	1469	43.5	0.008	49.2	0.015
≥ 16	878	49.0		53.6	
< 18	1649	44.3	0.061	49.8	0.074
≥ 18	698	48.4		53.4	
< 20	1806	45.1	0.334	50.4	0.156
≥ 20	541	46.7		52.3	
< 22	1938	45.2	0.405	50.5	0.200
≥ 22	409	46.4		52.3	
< 24	2049	45.4	0.579	50.9	0.611
≥ 24	298	46.0		50.1	

### Exploration for the optimal cut-off value of positive lymph nodes

**Fig 2:**
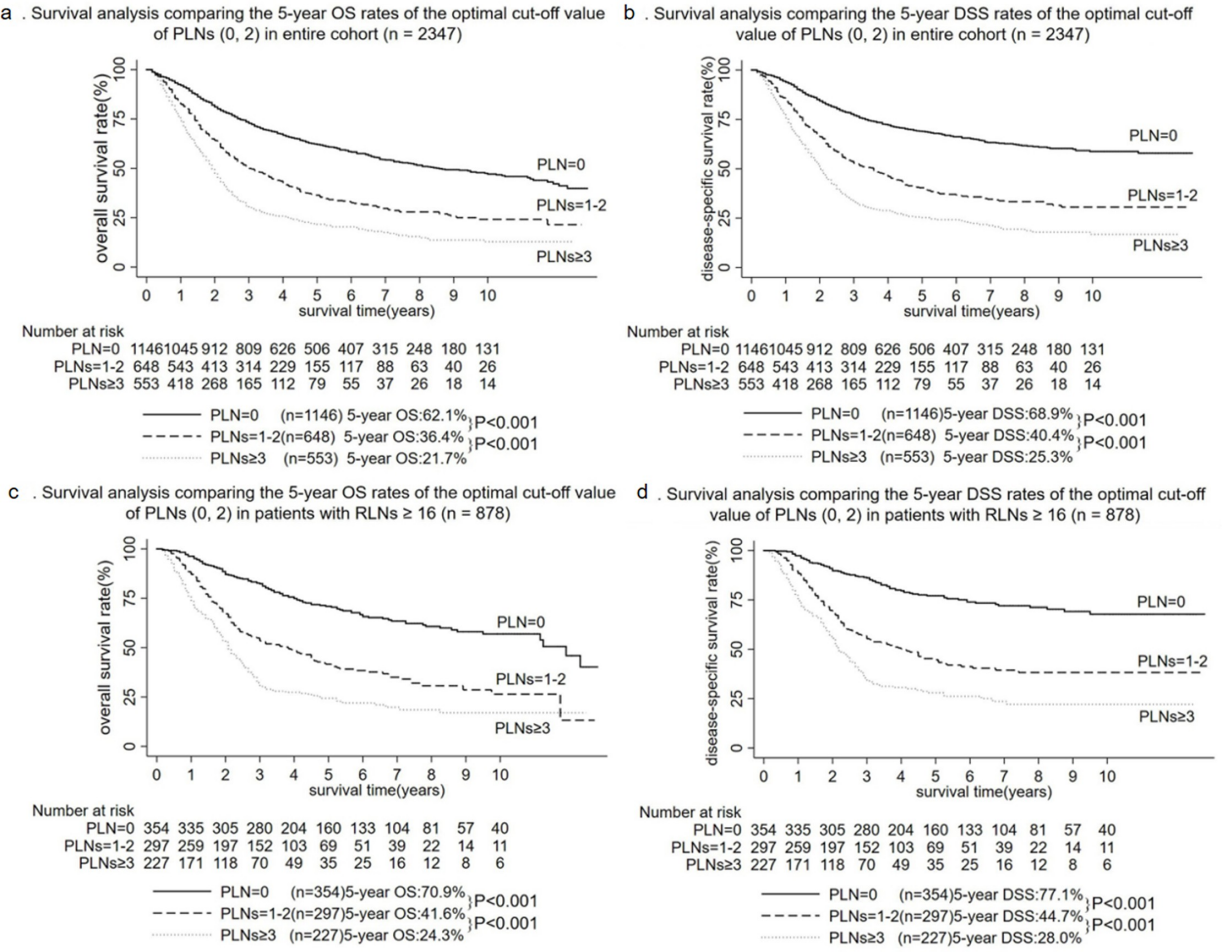
Survival analysis comparing the different numbers of PLNs. a: Survival analysis comparing the 5-year OS rates of the optimal cut-off value of PLNs (0, 2) in entire cohort (n = 2347). b: Survival analysis comparing the 5-year DSS rates of the optimal cut-off value of PLNs (0, 2) in entire cohort (n = 2347). c: Survival analysis comparing the 5-year OS rates of the optimal cut-off value of PLNs (0, 2) in patients with RLNs ≥ 16 (n = 878). d: Survival analysis comparing the 5-year DSS rates of the optimal cut-off value of PLNs (0, 2) in patients with RLNs ≥ 16 (n = 878).

The media numbers of PLNs were 1 (0–31) in the entire cohort (n = 2347) and 1 (0–31) in patients with RLNs ≥ 16 (n = 878). Whether in the entire cohort or in patients with RLNs ≥ 16, the optimal cut-off values of PLNs were 0 and 2 obtained by X-tile software. In the entire cohort (n = 2347), patients with PLN = 0 demonstrated significantly better 5-year OS and DSS rates (62.1% and 68.9%) compared to those with PLNs = 1–2 (36.4% and 40.4%; **Fig 2A** and **2B**; all P < 0.001), and patients with PLNs = 1–2 demonstrated significantly better 5-year OS and DSS rates (36.4% and 40.4%) compared to those with PLNs ≥ 3 (21.7% and 25.3%; **Fig 2A** and **2B**; all P < 0.001). In patients with RLNs ≥ 16 (n = 878), patients with PLN = 0 demonstrated significantly better 5-year OS and DSS rates (70.9% and 77.1%) compared to those with PLNs = 1–2 (41.6% and 44.7%; **Fig 2C** and **2D**; all P < 0.001), and patients with PLNs = 1–2 demonstrated significantly better 5-year OS and DSS rates (41.6% and 44.7%) compared to those with PLNs ≥ 3 (24.3% and 28.0%; **Fig 2C** and **2D**; all P < 0.001).

## Discussion

The numbers of RLNs and PLNs were important predictors of survival in patients with AVC [9–12], but it was controversial. Therefore, the end of this study was to explore the prognostic value of RLNs and PLNs for AVC and to determine the optimal point of the both through a national database.

The optimal cut-off value of RLNs supported by this study was 16. Adequate lymph node dissection may improve the prognosis of patients undergoing radical surgery for malignant tumors. Thus, routine lymph node dissection was recommended in AVC patients who underwent surgery [8], whereas there was some controversy over the specific number of RLNs [12, 13]. By comparing the 5-year OS and DSS rates of different cut-off values of RLNs in 2347 M0 patients with AVC who underwent surgical resection and lymph node dissection, the study found that the best cut-off values of RLNs were 18 in N0 cohort and 16 in N1 or entire cohort (**Table 2**). Overall, this study supported the optimal cut-off value of RLNs of 16 due to the highest 5-year OS rate (49.0%) and DSS rate (53.6%) and separation of survival curves between patients with RLNs ≥ 16 and RLNs < 16 in entire cohort (**Fig 1E**, P = 0.008; **Fig 1F**, P = 0.015), which was consistent with the results of a study by Falconi et al. [12]. Falconi et al. [12] found that AVC patients with RLNs ≥ 16 had a good prognosis. However, a single-center study from Europe supported an optimal cut-off value of RLNs of 12, whereas the sample size of the study was only 127 and the method of assigning cut-off values was not objective [13]. Moreover, it seemed feasible to examine at least 16 lymph nodes in patients with AVC because some studies reported that the median RLNs were around 16 [12, 14–16]. In addition, the prognosis of patients with malignant tumor may be better because more lymph nodes were removed. Therefore, this study recommended removing at least 16 lymph nodes.

The best cut-off values of PLNs recommended for this study were 0 and 2. Although there were many predictors affecting the prognosis of AVC, such as age [17], tumor size [10], and differentiation [1], lymph node status was recognized as a key factor affecting the prognosis of the patients [6, 10, 18–20]. The prognosis of AVC patients was poor as the number of positive lymph nodes increased [9, 10]. Bourgouin et al. [9] found that 5-year disease-free survival rates were statistically different among 55 AVC patients with PLN = 0, PLNs = 1–3, and PLNs ≥ 4. But the study of 2347 M0 patients with AVC found that the optimal cut-off values of PLNs were 0 and 2 whether in the entire cohort or in patients with RLNs ≥ 16. Moreover, a study including 1057 AVC patients who underwent surgical resection and had at least 12 lymph nodes examined found that the lymph node classification of AVC should be N0 (PLN = 0), N1 (PLNs = 1–2) and N2 (PLNs ≥ 3) [11], which were consistent with the results of our research. In summary, the best cut-off values of PLNs recommended for this study were 0 and 2.

This study had several limitations. Firstly, although the median number of RLNs in this series was 13, the number of RLNs in some patients was lower, which may hinder the full assessment of lymph node status and affect overall results. Secondly, since the SEER database lacked certain factors that affect the prognosis, such as surgical margins and specific sites of lymph node involvement, a multivariate analysis was not performed in this study, which may affect the results of this study. Thirdly, while we performed a comprehensive verification of the optimal cut-off value of PLNs calculated by software, any software had its shortcomings. New tools or methods for more comprehensive evaluation of optimal cut-off value may be produced in the future, which may affect the results of this study. Nevertheless, research based on a national database could provide greater generality to the results of this study.

## Conclusions

This study recommended that at least 16 lymph nodes will improve the prognosis of patients with AVC undergoing surgery. The best cut-off values of PLNs recommended for this study were 0 and 2, which may accurately stratify patients.

## Supporting information

S1 Checklist STROBE statement—checklist of items that should be included in reports of observational studies. (DOCX)
