# Supplementary material for: Prognostic importance of numbers of retrieved lymph nodes and positive lymph nodes for ampulla of vater cancer (AVC) in 2347 patients from the Surveillance, Epidemiology, and End Results (SEER) database
Source: PLoS One. 2021 Jan 15;16(1):e0244987. doi: 10.1371/journal.pone.0244987 (PMC7810317; doi:10.1371/journal.pone.0244987)
Supplement: S1 Checklist — (DOCX) [file pone.0244987.s001.docx]

S1 Checklist. STROBE Statement—checklist of items that should be included in reports of observational studies

|  | Item No. | Recommendation | Page  No. | Relevant text from manuscript |
| --- | --- | --- | --- | --- |
| **Title and abstract** | 1 | (*a*) Indicate the study’s design with a commonly used term in the title or the abstract | 2 | cohort study |
|  |  | (*b*) Provide in the abstract an informative and balanced summary of what was done and what was found | 2-3 | Results and Conclusions |
| Introduction | | | |  |
| Background/rationale | 2 | Explain the scientific background and rationale for the investigation being reported | 4 | Increasing evidence indicated that the numbers of retrieved lymph nodes (RLNs) and positive lymph nodes (PLNs) had a significant impact on the survival of patients with Ampulla of vater cancer (AVC). Due to the relative lack of disease, current studies about the effects of RLNs and PLNs on the prognosis of patients with AVC were mostly small sample sizes, and the optimal numbers of the both were controversial. |
| Objectives | 3 | State specific objectives, including any prespecified hypotheses | 4 | the cohort study aimed to explore the prognostic value of RLNs and PLNs for AVC and to determine the optimal point of RLNs and PLNs at the population level through a national database. |
| Methods | | | |  |
| Study design | 4 | Present key elements of study design early in the paper | 5 | To get the optimal cut-off value of RLNs, the survival analysis performed by Kaplan-Meier method (log-rank test) was comparing the 5-year OS and DSS rates of different cut-off values of RLNs in N0, N1 and entire cohorts, respectively, and it was used the 5-year survival rates and P values of different cut-off values of RLNs as line charts. |
| Setting | 5 | Describe the setting, locations, and relevant dates, including periods of recruitment, exposure, follow-up, and data collection | 4-5 | The data from the Surveillance, Epidemiology, and End Results (SEER) database was acquired from the SEER*Stat 8.3.6 Software (https://seer.cancer.gov/data/). A total of 2875 AVC patients who underwent surgical resection and lymph node dissection (RLNs ≥ 1) from January 2004 to December 2013 were identified by that primary site of tumor was C24.1-Ampulla of Vater, year of diagnosis was from 2004 to 2013, surgery of primary tumor was encoded from 20 to 90 (such as simple/partial surgical removal of primary site, total surgical removal of primary site, radical surgery), number of lymph nodes examined ≥ 1, diagnostic confirmation was positive histology, and type of follow-up expected was active follow-up. A number of 510 patients who < 18 years old or > 80 years old, died within 1 month, and had M1, MX, or NX diseases were excluded. Finally, 2347 AVC patients with M0 disease were included in the study. |
| Participants | 6 | (*a*) *Cohort study*—Give the eligibility criteria, and the sources and methods of selection of participants. Describe methods of follow-up  *Case-control study*—Give the eligibility criteria, and the sources and methods of case ascertainment and control selection. Give the rationale for the choice of cases and controls  *Cross-sectional study*—Give the eligibility criteria, and the sources and methods of selection of participants | 4-5 | The data from the Surveillance, Epidemiology, and End Results (SEER) database was acquired from the SEER*Stat 8.3.6 Software (https://seer.cancer.gov/data/). A total of 2875 AVC patients who underwent surgical resection and lymph node dissection (RLNs ≥ 1) from January 2004 to December 2013 were identified by that primary site of tumor was C24.1-Ampulla of Vater, year of diagnosis was from 2004 to 2013, surgery of primary tumor was encoded from 20 to 90 (such as simple/partial surgical removal of primary site, total surgical removal of primary site, radical surgery), number of lymph nodes examined ≥ 1, diagnostic confirmation was positive histology, and type of follow-up expected was active follow-up. A number of 510 patients who < 18 years old or > 80 years old, died within 1 month, and had M1, MX, or NX diseases were excluded. Finally, 2347 AVC patients with M0 disease were included in the study. The last follow-up time was November 2018. |
|  |  | (*b*) *Cohort study*—For matched studies, give matching criteria and number of exposed and unexposed  *Case-control study*—For matched studies, give matching criteria and the number of controls per case |  |  |
| Variables | 7 | Clearly define all outcomes, exposures, predictors, potential confounders, and effect modifiers. Give diagnostic criteria, if applicable | 5 | The data collected in this study included age at diagnosis, gender, race, marital status at diagnosis, tumor size, tumor grade, lymph node metastasis, number of RLNs and PLNs, adjuvant radiotherapy and chemotherapy. |
| Data sources/ measurement | 8* | For each variable of interest, give sources of data and details of methods of assessment (measurement). Describe comparability of assessment methods if there is more than one group | *5* | Overall survival (OS) and disease-specific survival (DSS) rates were calculated from diagnosis to death due to any reason and from diagnosis to death due to VAC, respectively. The last follow-up time was November 2018. To get the optimal cut-off value of RLNs, the survival analysis performed by Kaplan-Meier method (log-rank test) was comparing the 5-year OS and DSS rates of different cut-off values of RLNs in N0, N1 and entire cohorts, respectively, and it was used the 5-year survival rates and P values of different cut-off values of RLNs as line charts. The optimal number of PLNs was gained by X-tile software (Version 3.6.1, Yale University) and verified by the survival analysis. |
| Bias | 9 | Describe any efforts to address potential sources of bias | 5 | A number of 510 patients who < 18 years old or > 80 years old, died within 1 month, and had M1, MX, or NX diseases were excluded. |
| Study size | 10 | Explain how the study size was arrived at | 4-5 | The data from the Surveillance, Epidemiology, and End Results (SEER) database was acquired from the SEER*Stat 8.3.6 Software (https://seer.cancer.gov/data/). A total of 2875 AVC patients who underwent surgical resection and lymph node dissection (RLNs ≥ 1) from January 2004 to December 2013 were identified by that primary site of tumor was C24.1-Ampulla of Vater, year of diagnosis was from 2004 to 2013, surgery of primary tumor was encoded from 20 to 90 (such as simple/partial surgical removal of primary site, total surgical removal of primary site, radical surgery), number of lymph nodes examined ≥ 1, diagnostic confirmation was positive histology, and type of follow-up expected was active follow-up. A number of 510 patients who < 18 years old or > 80 years old, died within 1 month, and had M1, MX, or NX diseases were excluded. Finally, 2347 AVC patients with M0 disease were included in the study. |

Continued on next page

| Quantitative variables | 11 | Explain how quantitative variables were handled in the analyses. If applicable, describe which groupings were chosen and why | 5 | To get the optimal cut-off value of RLNs, the survival analysis performed by Kaplan-Meier method (log-rank test) was comparing the 5-year OS and DSS rates of different cut-off values of RLNs in N0, N1 and entire cohorts, respectively, and it was used the 5-year survival rates and P values of different cut-off values of RLNs as line charts. The optimal number of PLNs was gained by X-tile software (Version 3.6.1, Yale University) and verified by the survival analysis. |
| --- | --- | --- | --- | --- |
| Statistical methods | 12 | (*a*) Describe all statistical methods, including those used to control for confounding | 5 | the Kaplan-Meier method (log-rank test) |
|  |  | (*b*) Describe any methods used to examine subgroups and interactions | 5 | X-tile software |
|  |  | (*c*) Explain how missing data were addressed | 5 | A number of 510 patients who < 18 years old or > 80 years old, died within 1 month, and had M1, MX, or NX diseases were excluded. |
|  |  | (*d*) *Cohort study*—If applicable, explain how loss to follow-up was addressed  *Case-control study*—If applicable, explain how matching of cases and controls was addressed  *Cross-sectional study*—If applicable, describe analytical methods taking account of sampling strategy | 5 | Type of follow-up expected was active follow-up. |
|  |  | (*e*) Describe any sensitivity analyses | 5 | The survival analysis performed by Kaplan-Meier method (log-rank test) |
| Results | | | | |
| Participants | 13* | (a) Report numbers of individuals at each stage of study—eg numbers potentially eligible, examined for eligibility, confirmed eligible, included in the study, completing follow-up, and analysed | - | No applicable |
|  |  | (b) Give reasons for non-participation at each stage | - | No applicable |
|  |  | (c) Consider use of a flow diagram | Nothing | Nothing |
| Descriptive data | 14* | (a) Give characteristics of study participants (eg demographic, clinical, social) and information on exposures and potential confounders | 6 | Table 1. Demographics and clinical characteristics for AVC patients with M0 disease (n = 2347). |
|  |  | (b) Indicate number of participants with missing data for each variable of interest | 6 | Table 1. Demographics and clinical characteristics for AVC patients with M0 disease (n = 2347). |
|  |  | (c) *Cohort study*—Summarise follow-up time (eg, average and total amount) | 6 | the median follow-up was 39 (2-155) months |
| Outcome data | 15* | *Cohort study*—Report numbers of outcome events or summary measures over time | *6* | As of November 2018, 981 (41.8%) patients survived and 1366 (58.2%) patients died. |
|  |  | *Case-control study—*Report numbers in each exposure category, or summary measures of exposure |  |  |
|  |  | *Cross-sectional study—*Report numbers of outcome events or summary measures |  |  |
| Main results | 16 | (*a*) Give unadjusted estimates and, if applicable, confounder-adjusted estimates and their precision (eg, 95% confidence interval). Make clear which confounders were adjusted for and why they were included | 8 | Table 2. Survival analysis comparing the 5-year OS and DSS rates of different cut-off values of RLNs in N0, N1 and entire cohorts for AVC patients with M0 disease (n = 2347). |
|  |  | (*b*) Report category boundaries when continuous variables were categorized | 8 | Table 2. Survival analysis comparing the 5-year OS and DSS rates of different cut-off values of RLNs in N0, N1 and entire cohorts for AVC patients with M0 disease (n = 2347). |
|  |  | (*c*) If relevant, consider translating estimates of relative risk into absolute risk for a meaningful time period | Nothing | Nothing |

Continued on next page

| Other analyses | 17 | Report other analyses done—eg analyses of subgroups and interactions, and sensitivity analyses | 8-13 | Survival analysis |
| --- | --- | --- | --- | --- |
| Discussion | | | | |
| Key results | 18 | Summarise key results with reference to study objectives | 16 | This article recommended that at least 16 lymph nodes will improve the prognosis of AVC patients undergoing surgery. The best cut-off values of PLNs recommended for this study were 0 and 2, which may accurately stratify patients. |
| Limitations | 19 | Discuss limitations of the study, taking into account sources of potential bias or imprecision. Discuss both direction and magnitude of any potential bias | 15 | One limitation of this study was that although the median number of RLNs in this series was 13, the number of RLNs in some patients was lower, which may hinder the full assessment of lymph node status and affect overall results. Another disadvantage was that this study lacked certain factors that affect prognosis due to the SEER database, such as surgical margins, specific sites of lymph node involvement, which may affect the results of this study. |
| Interpretation | 20 | Give a cautious overall interpretation of results considering objectives, limitations, multiplicity of analyses, results from similar studies, and other relevant evidence | 16 | This article recommended that at least 16 lymph nodes will improve the prognosis of AVC patients undergoing surgery. The best cut-off values of PLNs recommended for this study were 0 and 2, which may accurately stratify patients. |
| Generalisability | 21 | Discuss the generalisability (external validity) of the study results | 15 | Research based on a national database could provide greater generality to the results of this study. |
| Other information | |  | | |
| Funding | 22 | Give the source of funding and the role of the funders for the present study and, if applicable, for the original study on which the present article is based | Nothing | No Funding. |

*Give information separately for cases and controls in case-control studies and, if applicable, for exposed and unexposed groups in cohort and cross-sectional studies.

**Note:** An Explanation and Elaboration article discusses each checklist item and gives methodological background and published examples of transparent reporting. The STROBE checklist is best used in conjunction with this article (freely available on the Web sites of PLoS Medicine at http://www.plosmedicine.org/, Annals of Internal Medicine at http://www.annals.org/, and Epidemiology at http://www.epidem.com/). Information on the STROBE Initiative is available at www.strobe-statement.org.
